# Development of A Rapid, Low-Cost Portable Detection Assay for Enterococci in Wastewater and Environmental Waters

**DOI:** 10.3390/microorganisms11020381

**Published:** 2023-02-02

**Authors:** Alka Rani Batra, Darren Cottam, Muriel Lepesteur, Carina Dexter, Kelly Zuccala, Caroline Martino, Leadin Khudur, Vivek Daniel, Andrew S. Ball, Sarvesh Kumar Soni

**Affiliations:** 1 ARC Training Centre for the Transformation of Australia’s Biosolids Resource, School of Science, RMIT University, Bundoora West, VIC 3083, Australia; 2 Environment Protection Authority Victoria, Centre for Applied Sciences, Ernest Jones Drive, Macleod, VIC 3085, Australia; 3 School of Science, RMIT University, Melbourne, VIC 3083, Australia

**Keywords:** *Enterococcus faecalis*, recombinase polymerase amplification, wastewater, lateral flow assay, water contamination

## Abstract

Waterborne diseases are known as a leading cause of illness and death in both developing and developed countries. Several pathogens can be present in contaminated water, particularly waters containing faecal material; however, routine monitoring of all pathogens is not currently possible. *Enterococcus faecalis*, which is present in the microflora of human and animals has been used as a faecal indicator in water due to its abundance in surface water and soil. Accurate and fast detection methods are critical for the effective monitoring of *E. faecalis* in the environment. Although conventional and current molecular detection techniques provide sufficient sensitivity, specificity and throughput, their use is hampered by the long waiting period (1–6 days) to obtain results, the need for expensive laboratory equipment, skilled personnel, and cold-chain storage. Therefore, this study aimed to develop a detection system for *E. faecalis* that would be simple, rapid, and low-cost, using an isothermal DNA amplification assay called recombinase polymerase amplification (RPA), integrated with a lateral flow assay (LFA). The assay was found to be 100% selective for *E. faecalis* and capable of detecting rates as low as 2.8 × 10^3^ cells per 100 mL from water and wastewater, and 2.8 × 10^4^ cells per 100 mL from saline water. The assay was completed in approximately 30 min using one constant temperature (38 °C). In addition, this study demonstrated the quantitation of *E. faecalis* using a lateral flow strip reader for the first time, enhancing the potential use of RPA assay for the enumeration of *E. faecalis* in wastewater and heavily contaminated environmental waters, surface water, and wastewater. However, the sensitivity of the RPA-LFA assay for the detection of *E. faecalis* in tap water, saline water and in wastewater was 10–1000 times lower than that of the Enterolert-E test, depending on the water quality. Nevertheless, with further improvements, this low-cost RPA-LFA may be suitable to be used at the point-of-need (PON) if conjugated with a rapid field-deployable DNA extraction method.

## 1. Introduction 

Globally, in 2020, approximately 2 billion people (26%) lacked safe drinking water, 3.6 billion people (46%) lacked safely managed sanitation—meaning access to a toilet or latrine that leads to treatment or safe disposal of excreta, and 2.3 billion people (29%) did not have access at home to a handwashing facility with soap and water [1]. Waterborne diseases represent a serious health burden worldwide due to the presence of faecal contamination and human enteric pathogens in water. Water released from wastewater treatment plants potentially plays an important role in the transmission of waterborne diseases because if poorly treated, faecal-contaminated water can be re-introduced into a body of water, applied to land, or can be re-used directly for irrigation purposes [2,3,4]. Treated wastewater can contain high microbial numbers even after primary, secondary and tertiary treatment processes [5,6]. Therefore, monitoring of water released from wastewater plants is crucial in managing waterborne diseases [7]. 

Microbial contamination in wastewater includes bacteria, viruses, helminth, protozoa etc. [5]; however, due to cost and time limitations, it is difficult to identify and quantify all of the organisms. Therefore, faecal indicator bacteria (FIB) are used; *Escherichia coli* is the most commonly used FIB in most countries for the assessment of water quality [7,8,9]. However, Enterococci, previously known as faecal streptococci, are also included in the list of FIB in water by the United States Environmental Protection Agency (USEPA). Enterococci are members of the human and animal gut microbiome; they are present predominantly in warm-blooded animals (10^5^–10^7^ per gram of faeces) [10,11]. Though detection of all *Enterococcus* sp. has limited significance in differentiating faecal/non-faecal contamination in water due to their omnipresence, their correlation with adverse health outcomes in marine waters has led to their widespread use as an indicator of faecal pollution and water quality globally. Recent studies report the use of *E. faecalis* as the most suitable FIB in water [11]. 

A sensitive and specific diagnostic test is critical for monitoring *E. faecalis* in recycled water and more generally in water quality monitoring. The current standard methods for detection of *E. faecalis* include sample culturing (serial dilution or membrane filtration followed by selective plate isolation), the Enterolert-E test, and polymerase chain reaction (PCR). These laboratory-based methods are largely accurate, provide sufficient sensitivity, specificity, and are high-throughput [12]. However, their use is hampered by costs associated with sample collection and a significant time (up to 6 days) for analysis [13,14]. This turnaround time can result in outdated advice being provided to the public in recreational water quality monitoring programs. The same challenges are experienced by water authorities warning the public about sewer spills to waterbodies. In addition, receiving results more than 24 h after sampling also makes investigating the cause of pollution difficult, as a pollution source may no longer be present. Therefore, the objective behind this research is to attempt to develop a potential rapid method which can be used by the environment sectors at the point-of-need (PON) for the timely diagnosis of contamination in water. To achieve this objective, we developed and evaluated an isothermal DNA amplification technique for *E. faecalis* in water and wastewater which is a rapid, simple, and specific detection assay.

To achieve the objective, recombinase polymerase amplification (RPA) was used in this study, which is a novel isothermal DNA amplification technology developed by Piepenburg et al. [15] and manufactured by TwistDX (a company based in Cambridge, UK). Requiring only constant ambient temperatures [16], it has been used as a versatile alternative to polymerase chain reaction (PCR)-based technologies for the development of rapid nucleic acid detection assays for several important pathogens [14,17,18,19]. The RPA technology is reported as a sensitive, specific technique and importantly overcomes many obstacles existing in the currently used routine molecular diagnostics [16,18,20,21]. The technology offers a rapid, robust, high-throughput, low-energy approach using readily portable equipment, with results available using field-friendly detection devices [18,22]. The technique has already been utilised for the detection of specific pathogens by our research group, such as *Ascaris* sp. and *E. coli* O157:H7 [23,24,25,26]. 

Recombinase polymerase amplification products can be visualised by a variety of detection methods, such as agarose gel electrophoresis (AGE), real-time quantitative fluorescence and lateral flow strips [17,23,27,28]. However, AGE requires time-consuming protocols and suffers from low sensitivity. Real-time, quantitative fluorescence overcomes this limitation, but requires multiple reaction steps, well-equipped facilities, and trained technicians [28]. Lateral flow (LF) strips based on oligochromatographic testing only require 10 min to achieve a valid result [23,29]. Lateral flow strips are popular since they can establish an affordable, sensitive, specific, user-friendly, and rapid visual detection assay for trace target detection [30,31]. For the visualisation of LF strips, gold nanoparticles are an ideal label material with several advantages. They are amenable to detection by multiple methods, stable under different assay conditions, and available commercially at low cost [17]. Colloidal gold nanoparticles coated on the LF strips can produce visible results, while allowing for solutions filtered through a nitrocellulose membrane without blockage. Therefore, LF combined with colloidal gold labelling has been extensively used for the qualitative or semi-quantitative detection of various analytes by the naked eye or with a simple strip reader [32,33,34].

The RPA combined with a rapid lateral flow detection system was recently reported by Zhu et al. to identify the presence of *E. faecalis* in clinical samples [35,36,37,38]. Further, Yin et al. reported the use of RPA in a 3D printed microfluidic chip to detect SARS-CoV-2 and other pathogens in wastewater [39]; however, no reports have detailed the development and application of the RPA assay to detect *E. faecalis* from environmental waters. The novelty of this work, therefore, is the development of an RPA-LFA validated for the rapid and specific detection of *E. faecalis* from water, wastewater, and saline. This technology has a fast turnaround time and the potential to deliver results at the point of sample collection.

## 2. Material and Methods 

### 2.1. Samples

Wastewater samples (20 L) were collected in triplicate in sterile polypropylene containers from two municipal wastewater treatment plants (Lang Lang and Koo Wee Rup, Melbourne, Victoria, Australia), operated by South East Water. Samples were stored on ice during transportation and in the laboratory until processing. The wastewater samples were pre-checked for presence of enterococci using the Enterolert-E test which is further discussed in Section 2.4. 

### 2.2. Bacterial Isolates

Pure culture of *Enterococcus faecalis* NCTC 12201, *Enterococcus faecium* (strain unknown), *Staphylococcus aureus* ATCC 6538, *Streptococcus pneumoniae* (strain unknown), *Bacillus cereus* ATCC 11778, *Pseudomonas aeruginosa* ATCC 27853, *Escherichia coli* O157:H7 ATCC 43895, *Salmonella typhimurium* ATCC 14028, *Shigella dysenteriae* NCTC 4837, and *Escherichia coli* K-12 NCTC 10538 were obtained from the Microbial Culture Collection Centre, RMIT University. *E. faecalis* was used as the target organism (indicator bacteria) for this study. The other bacterial isolates were used to validate the primers for cross-reactivity and for comparison with the Enterolert-E test (Enterolert E, IDEXX, Rydalmere, New South Wales, Australia), a benchmark standard for the detection of enterococci. 

All bacterial strains were cultivated (sub-cultured) in nutrient broth (No 3, Sigma-Aldrich, Merck KGaA Darmstadt, Germany) at 37 °C with shaking at 180 rpm. Growth was monitored by measuring the optical density at 600 nm until an OD_600_ of 0.5–0.6 was reached, at which point bacterial DNA was extracted.

### 2.3. Standard Plate Count for Enterococcus faecalis

A series of known concentrations of *E. faecalis* was prepared to assess the sensitivity of the RPA assay. *E. faecalis* was sub-cultured in nutrient broth (NB) and incubated at 37 °C overnight. After 24 h, 10-fold serial dilutions were prepared ranging from 10^−1^ (10^6^ organism/100 mL) to 10^−7^ (10^0^ organism/100 mL). One hundred µL of each dilution was plated on nutrient agar (NA) plates and incubated at 37 °C overnight. The number of organisms/100 mL was then enumerated. In addition, a standard plate count was used to determine the number of enterococci present naturally in wastewater samples. All results are expressed as organisms/100 mL.

### 2.4. Enterolert-E Test

The Enterolert-E test, the standard method (ISO 7899-1) for enterococci, was performed to compare the sensitivity with the RPA-LFA assay (elaborated in Section 2.9). The Enterolert-E test determines the presence/absence and numbers of enterococci based on the IDEXX Defined Substrate Technology (DST) nutrient indicator. This nutrient indicator produces fluorescence when metabolised by enterococci. The test was conducted according to the manufacturer’s instructions using the Quanti-Tray/2000 (Enterolert E, IDEXX, Rydalmere, New South Wales, Australia). The different dilutions of *E. faecalis* were prepared and artificially spiked into enterococci negative samples (tap water, saline, and wastewater). The inoculated samples were sealed in plates and incubated at 41 °C for 18–24 h. The plates were observed after 24 h under a UV illuminator. Blue illuminated wells were counted as positive for *E. faecalis* and the most probable number (MPN) calculated following the manufacturer’s instructions. Although MPN is calculated using the Enterolert-E test, selective plate isolations were used throughout the experiments to count the number of bacteria. For simplification, the bacterial counts obtained by all methods described here are expressed as the number of organisms/100 mL.

### 2.5. Total DNA Extraction from Selected Bacteria

Genomic DNA was extracted from all bacterial strains listed in Section 2.2 (approximately 100 mg bacterial cell pellets resuspended in 200 µL of 1× phosphate buffered saline (PBS)) using Quick DNA^TM^ Soil/Faecal Microbe Miniprep kits (Zymo Research, Irvine, CA, USA), quantified using a Qubit 2.0 fluorometer (Invitrogen, Waltham, MA, USA), and stored at −20 °C for further use. In addition, DNA concentrations and its purity were confirmed by the ratio of absorbance at 260 and 280 nm using Nanodrop spectrophotometry. 

### 2.6. Designing and Screening of RPA Primers

To design the specific primers, species-specific genes with highly conserved sequence regions for *E. faecalis* were chosen. The genes encoding *tuf* (elongation factor Tu) [40], *rpo*A, and *rpo*B (RNA polymerase subunits A and B respectively) [41,42,43] have previously reported to be suitable targets for enterococci identification. Therefore, primers encoding these conserved regions were prepared. The primers for the target genes were designed using NCBI’s primer-BLAST and PrimedRPA tools as per the instructions given by TwistDX (TwistDx, UK). Recombinase polymerase amplification primers were designed between 30 and 35 nucleotides long with 30–60% GC content and 100–300 base pair (BP) amplicon size. The OligoAnalyzer tool (IDT) was used to analyse the cross-binding potential of primers specific to other bacteria in the enterococci genus. Based on in silico analysis, primers that had less than 6 overall mismatches to non-targets were removed and the chosen primer sets were further screened for in vitro analysis. Table 1 listed the primers and probes optimised for the target genes. All primers were purchased from Integrated DNA technologies (IDT, Boronia, Victopria, Australia).

### 2.7. Recombinase Polymerase Amplification-Agarose Gel Electrophoresis (RPA-AGE)

Primer screening (in vitro) was performed by amplifying the target genes using TwistDx Basic RPA kits (TwistDx, Maidenhead, UK) followed by purification of amplicons using Isolate II PCR and Gel kits (Bioline, London, UK). End-point analysis was performed using agarose gel electrophoresis (AGE); 2% agarose gels were used for visualisation of the bands. Amplification was optimised for temperature (35 °C to 42 °C) and time (5 to 40 min) after selection of the primers based on the observation of the bands (180–200 bp). Briefly, the reaction was performed in 50 µL volume containing 29.5 µL rehydration buffer (provided in the kit), 2.4 µL of both forward and reverse primer sets, 11.2 µL nuclease-free water, and 2 µL of genomic DNA template. The mixture was added to the freeze-dried reaction pellet (provided in the kit) and mixed with a pipette. An aliquot (2.5 µL) of magnesium acetate (14 mM) was added to the lid of the reaction tube and centrifuged briefly. The tubes were placed in a thermal cycler at 38 °C for 20 min. The amplified RPA product was purified using Isolate II PCR and Gel kit (Bioline, London, UK) and subsequently visualised on a 2% agarose gel with 100 bp molecular marker (Thermofisher Scientific, Waltham, MA, USA). The negative control consisted of the reaction mix excluding the genomic DNA. To avoid contamination, separate locations within the laboratory were used to set-up and purify the reactions. 

### 2.8. Recombinase Polymerase Amplification-Lateral Flow Assay (RPA-LFA)

Following primer optimisation using agarose gel electrophoresis as the end point analysis, a prototype was developed using RPA coupled with the lateral flow assay. The assay was performed as per the TwistDx nfo kit (TwistDx, Maidenhead, UK) instructions. One of the primer sets (reverse) was labelled using biotin at the 5′ end and the *nfo* (endonuclease 4) probe was designed having a 5′-FAM and tetrahydrofuran (THF) inserted. A C3 spacer which blocks the extension of DNA polymerase was added to the 3′ end of the probe. 

The RPA–*nfo* reaction was performed in 50 µL by adding 29.5 µL rehydration buffer (from the kit), 2.1 µL of each forward and reverse primer, 0.6 µL *nfo* probe, 11.2 µL nuclease-free water, 2.5 µL magnesium acetate, and 2 µL genomic DNA. The lyophilised enzyme pellet (provided in the kit) was dissolved by adding 45.5 µL of prepared master mix into 200 µL RPA reaction tubes. Subsequently, 2 µL of DNA template was added to the RPA tube prior to adding 2.5 µL magnesium acetate into the lids of the tube. The lids were closed, and the tubes centrifuged briefly before incubation at 38 °C for 20 min in a thermal cycler. 

PCRD lateral flow dipsticks (Abingdon Health, London, UK) were used to visualise the amplified product via the presence/absence of coloured bands. An aliquot (6 µL) of amplified product was diluted with 84 µL of PCRD reaction buffer (provided in the kit) and 75 µL from the mixture was loaded onto the sample application area of the dipstick. The dipstick was placed on the bench and left undisturbed for the capillary flow of the sample (upward stream). The appearance of the bands at the control and test lines were recorded as appropriate within 10 min (bands appear in 2–3 min). The presence of coloured bands in both the control and test lines was deemed a positive result, while the presence of bands in the control line alone indicated a negative result. 

### 2.9. Selectivity and Sensitivity of Recombinase Polymerase Amplification (RPA)

In the validation step, selectivity tests were performed for both RPA-AGE and RPA-LFA as per the protocol described in Section 2.7 and Section 2.8. The other non-target bacteria included were *E. faecium*, *S. aureus*, *S. pneumoniae*, *B. cereus*, *P. aeruginosa*, *E. coli* O157:H7, *S. typhimurium*, *S. dysenteriae*, and *E. coli* K-12. In addition, water was used to include a non-template control (NTC).

Sensitivity evaluation was also conducted for both RPA-AGE and RPA-LFA using 10-fold serial dilutions ranged from 2.8 × 10^6^ CFU/100 mL to 2.8 CFU/100 mL. The sensitivity was checked with pure culture of *E. faecalis* in tap water, wastewater (collected from Lang Lang and Koo Wee Rup), and saline solution. The wastewater samples were pre-confirmed for the presence of enterococci using the Enterolert-E test as in Section 2.1 and Section 2.4. The enterococci-positive wastewater was sterilised before assessing the sensitivity of the RPA assay if applicable. Uninoculated tap water, saline and wastewater were included as NTC in the sensitivity experiments. A band of the expected amplicon size was considered positive on the gel and a coloured band at test line was considered positive in LFA. 

### 2.10. Quantification of the Recombinase Polymerase Amplification-Lateral Flow Assay (RPA-LFA) Using the Benchtop Reader

An RPA-LFA reader was designed and purchased from Creative Diagnostics, NY, USA (https://www.creative-diagnostics.com, accessed on 7 February 2021) to quantitate the intensity of the test bands. This immunochromatographic reader analyses the colloidal gold nanoparticles on the control and test line to produce an arbitrary unit. Tenfold serially diluted *E. faecalis* was used to generate a standard curve between arbitrary units versus the used cells concentration. A known cell concentration of pure *E. faecalis* was used to cross-confirm the functioning of the device. For operating the device, the manufacturer’s instructions were followed. 

## 3. Results and Discussion

### 3.1. DNA Extraction and Selection of the Primers

Contaminated water is the leading cause of many respiratory and enteric diseases. Identification of faecal indicator bacteria (FIB) is one common approach used to assess the level of microbial contamination before release of treated water into the environment [3,44]. The FIB are usually the commensal microflora of the gut such as *E. coli*, *Clostridium perfringens*, *S. aureus* and *Enterococci*, among others [2,7]. The detection of *E. faecalis* has become crucial for water quality monitoring since the *Enterococci* group was classified under the list of faecal indicators by USEPA [11]. Several standard methods have been approved for *E. faecalis* enumeration from water including Enterolert and Enterolert-E [44]. However, there is an urgent need for the development of a rapid point-of-need (PON) molecular detection approach for *E. faecalis* from water and wastewater. Thus, in this study, we aimed to develop, assess, and validate the potential of an isothermal DNA amplification approach known as recombinase polymerase amplification (RPA) integrated with a lateral flow assay (LFA) to be used by the water and environment sector for the rapid and simple detection of *E. faecalis* at the PON. 

Initially, the DNA was successfully isolated from a range of bacterial strains; the purity of the DNA was checked by spectrophotometry. The DNA from all bacterial strains exhibited an OD_260/280_ between 1.8 and 1.9, suggesting a high-quality product, with DNA concentrations higher than 100 ng/µL. 

**Figure 1:**
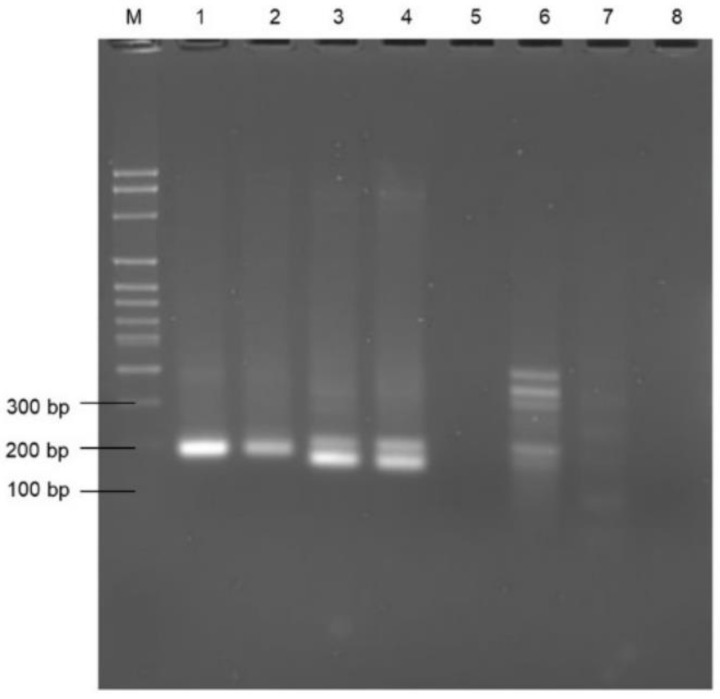
Screening of primers: Lanes 1 and 2—Ent rpoB F1/R1, Lanes 3 and 4—Ent rpoA F1/R1, Lane 5 and 6—Enttuf F1/R1, Lane 7 and 8—Enttuf F2/R2. Lane M—100 bp molecular ladder. (Note: primer concentration is −10 µM, template—2 µL, assay temperature and time—38 °C for 20 min).

**Figure 2:**
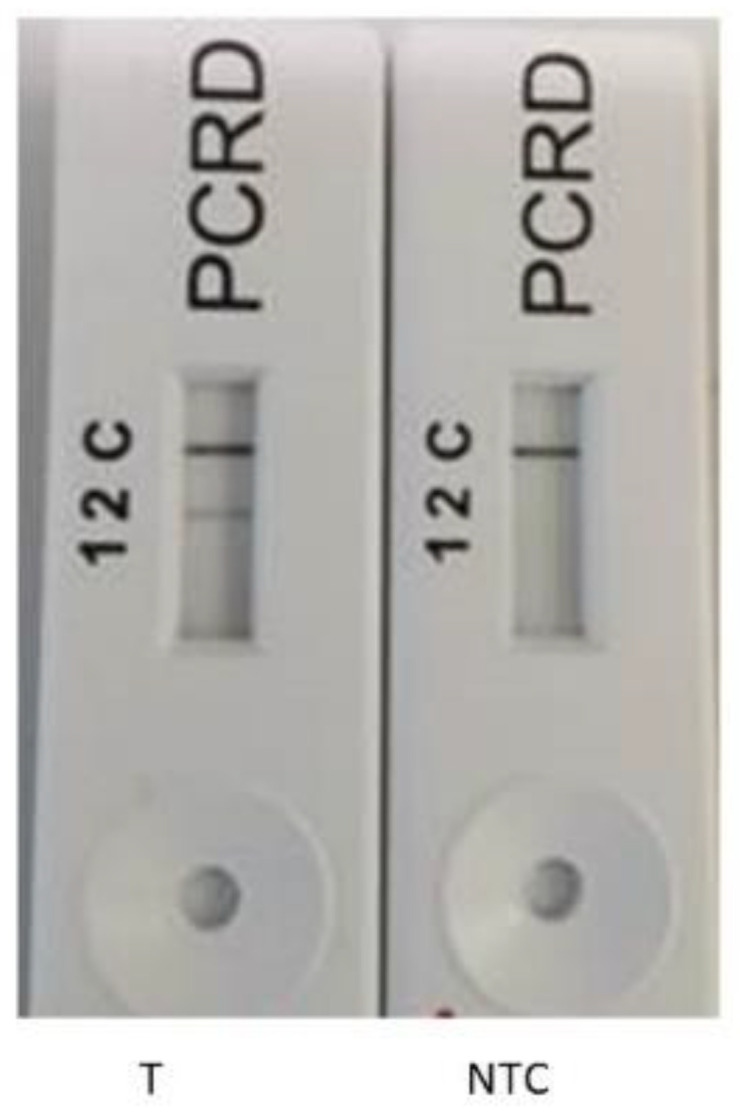
Lateral flow assay: RPA-LFA using the Ent rpoB F1/R1 primer set with positive template *E. faecalis* DNA (T), and non-template control (NTC).

The PrimedRPA software was selected due to its previously reported specification for RPA primers; it was used with all filters set by default [23]. The primers were then screened for the amplification of the genomic DNA of *E. faecalis*. The primers for the *rpoB* gene were selected for further experiments based on the expected amplicon size (approx. 185 bp) on the agarose gel (Figure 1, lanes 1 and 2). Consequently, the optimum temperature and time was chosen as 38 °C for 20 min following temperature and time optimisation (gel images not shown). The nfo probe was designed to develop the RPA-LFA combined with PCRD lateral flow test strips. The PCRD LF strips were designed to detect the appropriately labelled amplification product. The LF assay has three reaction lines (Figure 2): line 1 detects DIG/Biotin labelled amplicons to produce a coloured line; line 2 detects FAM/Biotin or FITC/Biotin labelled amplicons; and line 3 (C-line) is the control line to check the flow of the liquids. In this study, FAM/Biotin labelling was used; therefore, test line 2 is expected to produce colour for positive samples, whereas no colour is expected at test line 1 as no DIG/Biotin labels were used in this study. In the initial experiments, the lateral flow test was carried out with the target (*E. faecalis*) and non-template control (NTC) only. Using the primer set Ent rpoB F1/R1, a band was observed at the test line 2 for the positive target template only, whereas no coloured test line 2 was seen in the NTC (Figure 2). For both target and NTC, the control line was prominent, confirming the accurate working of the assay. The results confirm the ability of the primer set Ent rpoB F1/R1 to amplify the DNA of *E. faecalis*. All subsequent work was conducted using this primer set. The *rpoB* gene has been previously reported for its high identification potential over other genes, such as *tuf*, *recA*, and *sodA* etc. [43]. 

### 3.2. Selectivity Assessment of the RPA Using Selected Primer Set 

**Figure 3:**
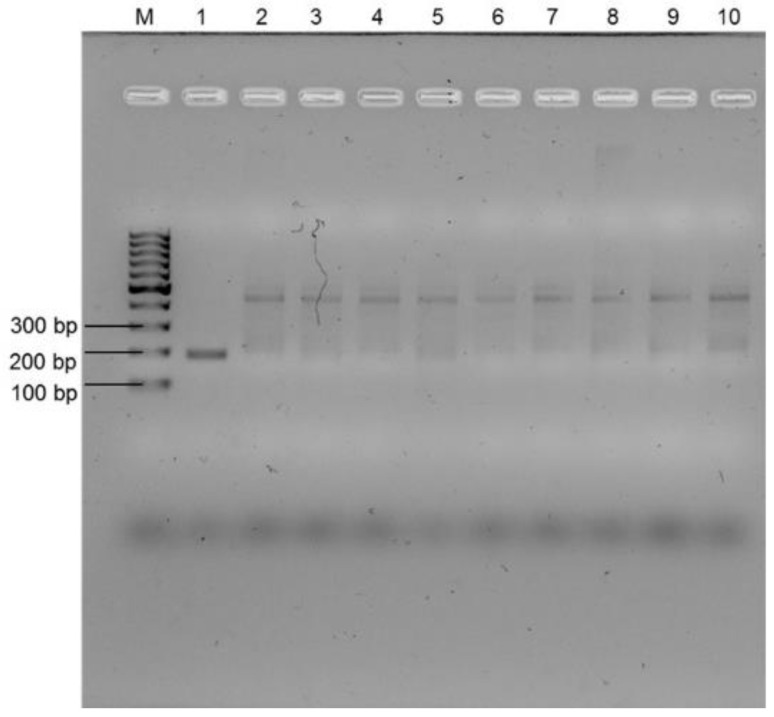
Specificity validation of Ent F1/R1 primer set using RPA-AGE. Lane M: 100 bp molecular marker, Lanes 1 to 10: *E. faecalis*, *E. faecium*, *Staphylococcus aureus*, *Streptococcus pneumoniae*, *Bacillus cereus*, *Pseudomonas aeruginosa*, *E. coli* O157:H7, *Salmonella typhimurium*, *Shigella dysenteriae*, *E. coli* K-12.

Further assessment of the selected primers was carried out by determining the specificity of the selected primers (Ent rpoB F1/R1) using DNA extracted from a range of other bacteria. Initially, the specificity validation test was performed using agarose gel electrophoresis (AGE). Figure 3 confirms that the primers amplified the expected amplicon size (approx. 185 bp) for *E. faecalis* only (Lane 1 in Figure 3). No similar sized amplicon was observed for any other bacterial species, including another enterococcus, *E. faecium*. However, in the gel (Figure 3), amplification of a band of approximately 700 bp size was seen for all other non-target organisms. This could be due to the primer binding to a non-targeted region. Figure S1 A shows no amplification when a non-template control using water was performed, which proves no primer-dimer formation. Having confirmed the specificity of the assay using AGE, the lateral flow assay (LF) was used to further assess the specificity of the primer. No coloured test line was observed for *E. faecium* and other Gram-positive and Gram-negative bacteria (Figure S1B), indicating that the designed assay and probe were 100% selective for the detection of *E. faecalis* without cross-amplifying with DNA from any other non-target bacteria. Overall, Figure 3 and Figure S1 confirm that the primers were specific for *E. faecalis* detection using RPA-EF and RPA-LFA. The enhanced selectivity of RPA-LFA over RPA-AGE to detect *E. faecalis* is due to the use of the nfo probe which binds to the specific amplified region of the template [19,45]. The 100% specificity of the RPA-LFA assay was also previously reported for the detection of *E. coli* O157:H7 from water [23]. The high selectivity of the RPA-LFA suggests the use of this assay for rapid PON detection method for *E. faecalis*, and could potentially be developed for antibiotic resistant *E. faecalis* from wastewater or other water resources [46].

### 3.3. Sensitivity of the Recombinase Polymerase Assay and Impact of Environmental Water Characteristics

**Figure 4:**
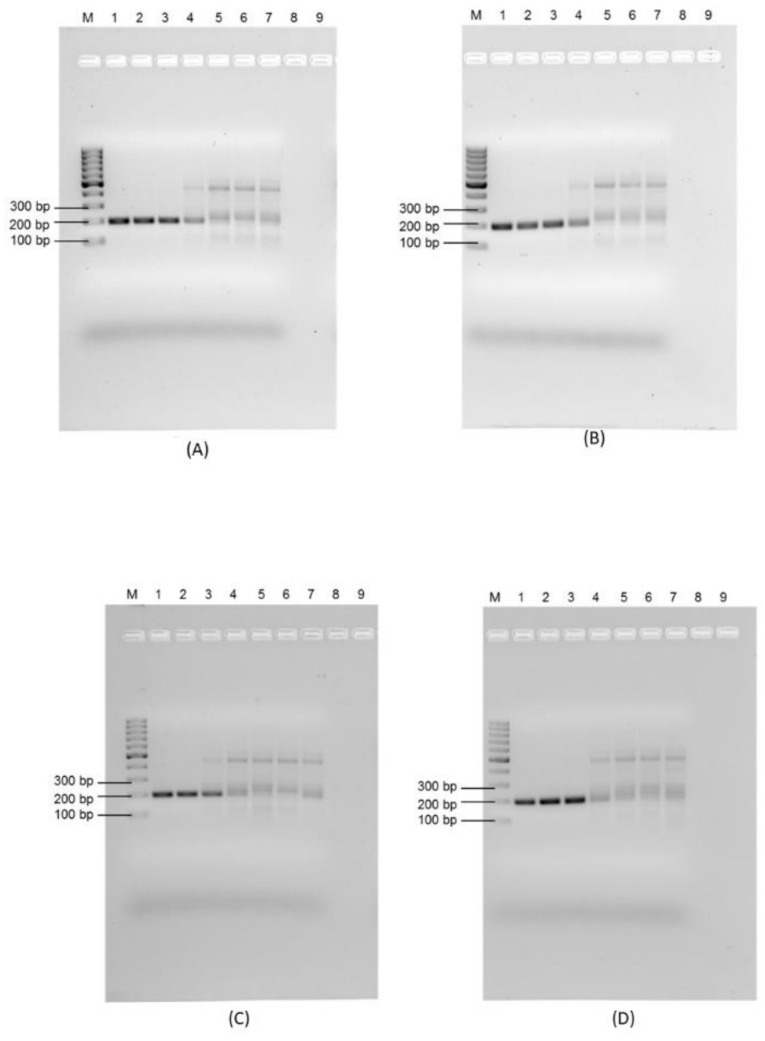
Sensitivity of RPA assay for detection of *E. faecalis* using (**A**) tap water; (**B**) Koo Wee Rup wastewater; (**C**) Lang Lang wastewater; (**D**) saline. Well M: molecular marker; Wells 1 to 7: 2.8 × 10^6^ CFU/100 mL, 2.8 × 10^5^ CFU/100 mL, 2.8 × 10^4^ CFU/100 mL, 2.8 × 10^3^ CFU/100 mL, 2.8 × 10^2^ CFU/100 mL, 2.8 × 10^1^ CFU/100 mL, 2.8 CFU/100 mL.

The impact of environmental waters on the sensitivity of the RPA assay was assessed using a pure culture of *E. faecalis* which was added to tap water, saline water, and wastewater, none of which contained any background enterococcal population. Plate isolation was first used to determine the actual number of enterococci that were prepared for inoculation (2.80 × 10^8^ organisms/mL). Using RPA-AGE, a clear dark band of the expected amplicon size was considered positive. The sensitivity limit of RPA-AGE was found to be 2.8 × 10^3^ organisms/100 mL for tap water (Figure 4A) and Koo Wee Rup wastewater (Figure 4B), and 2.8 × 10^4^ organisms/100 mL for Lang Lang wastewater (Figure 4C) and saline water (Figure 4D).

The assay was repeated using RPA with the lateral flow assay (Figure S2). Figure S2 shows that the limit of detection using RPA-LFA was 2.8 × 10^3^ organisms/100 mL for tap water (Figure S2A), which also indicates the sensitivity of the assay for the positive control, 2.8 × 10^3^ organisms/100 mL for Koo Wee Rup wastewater (Figure S2B), 2.8 × 10^4^ organisms/100 mL for Lang Lang wastewater (Figure S2C), and 2.8 × 10^3^ organisms/100 mL for saline water (Figure S2D). The results are based on the visual detection of the bands pictured in Figure 4. Both RPA-AGE and RPA-LFA were found to be equally sensitive for tap water, Koo Wee Rup, and Lang Lang wastewater, detecting as low as 10^3^ organisms/100 mL, which contradicts the results of a previous report [23], in which RPA-LFA was reported to be 10 times more sensitive than RPA-AGE in the detection *E. coli* O157:H7. However, 10 times more sensitive RPA-LFA results compared to RPA-AGE for saline water supports the findings of the same study [23]. The detection efficiency was more sensitive than the RPA-LF method developed for *E. faecalis* from clinical isolates [36], which could be due to the complexity difference of the samples. The detection efficiency was comparable to previously reported real-time PCR assays (10^4^–10^5^ organisms/100 mL) in faecal-contaminated water; however, the RPA-LFA was completed in 30 min without using a thermal cycler [47,48]. 

Having established the sensitivity of the RPA-AGE and RPA-LFA techniques to detect inoculated *E. faecalis* in tap water, saline water, and wastewater, a comparison was undertaken between the sensitivity of the RPA-based assays and that of the Enterolert-E test (Table 2). 

MPNs were calculated in a range of dilutions from 2.8 × 10^1^ to 2.8 × 10^2^ organisms/100 mL for tap water. Above that concentration, all wells were positive resulting in an infinite number of MPNs being counted. No positive wells were observed at a concentration of 2.8 organisms/100 mL. As expected, there were no positive results when tap water not spiked with *E. faecalis* was tested (Table 2). Most probable Numbers were calculated in a range of dilutions from 2.8 × 10^2^ to 2.8 × 10^3^ organisms/100 mL for saline water. No positive wells were observed at or below a concentration of 2.8 × 10^1^ organisms/100 mL. There were no positive results when saline water without spiking with *E. faecalis* was tested (Table 2). 

A comparison between the sensitivity of the RPA system and the Enterolert-E test based on the inoculation of known concentrations of *E. faecalis* in tap water and saline water showed that the Enterolert-E test was 10–1000 times more sensitive that the RPA system (either RPA-AGE or RPA-LF). The removal of inhibitors from faecal-containing samples, such as wastewater, may increase the sensitivity of the RPA [47]. The results suggest that the RPA system lacks the sensitivity of the Enterolert-E test and, therefore, would be unsuitable as a direct replacement for the laboratory-based Enterolert E-test. A minimum of 10^3^ cells per 100 mL would be required to enable detection for the RPA system. However, the potential use of the RPA-LFA system for the rapid detection of enterococci warranted further investigation.

For Koo Wee Rup wastewater tested using the Enterolert-E test, the artificially non-spiked wastewater sample was used as control and was found to be negative for the presence of enterococci. Using the Enterolert-E test, the MPNs were calculated in a range of dilutions ranging from 2.8 × 10^0^ to 2.8 × 10^1^ organisms/100 mL (Table 2). The variable sensitivity from different samples can be explained based on previous reports on the possibilities of over- and underestimation of FIB using an MPN count system [44]. 

**Figure 5:**
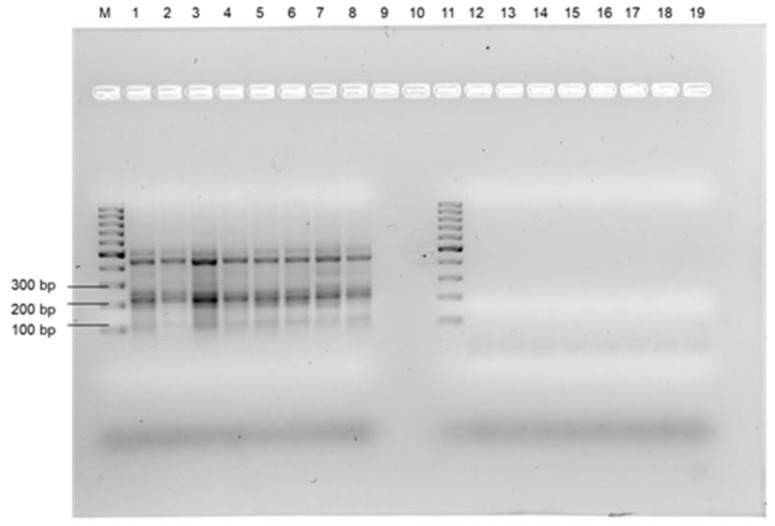
Confirmation of the presence of *E. faecalis* in Lang Lang wastewater. Lanes M and 11: molecular marker, Lanes 1–8: RPA-AGE of Lang Lang wastewater neat, 10^−1^, 10^−2^, 10^−3^, 10^−4^, 10^−5^, 10^−6^, 10^−7^. Lanes 12–19: PCR of Lang Lang wastewater neat, 10^−1^, 10^−2^, 10^−3^, 10^−4^, 10^−5^, 10^−6^, 10^−7^.

For the Lang Lang wastewater, a natural enterococcus population was found with the Enterolert-E test; therefore, Enterolert-E test, RPA-AGE, RPA-LFA, PCR and plate culture assays were performed using tenfold serial dilutions of uninoculated wastewater samples. The Enterolert-E test gave positive detection for enterococci up to the 10^−5^ dilution for the Lang Lang wastewater (number are given in Table 2), suggesting a natural enterococci population of 0.7–3.1 × 10^5^ organisms/100 mL. In contrast, no positive results were observed for the presence of *E. faecalis* using either of the molecular assays, i.e., PCR, RPA-AGE, and RPA-LF, suggesting that the wastewater was not contaminated with *E. faecalis*, but with other enterococci (Figure 5 and Figure S3). The presence of enterococci was confirmed using selective plates. The primers selected for the work showed high specificity to *E. faecalis* and very low, if any, specificity to other enterococci species that gave positive results using the Enterolert-E test (Table 3). Therefore, the RPA-LFA developed in this project was specific for the detection of *E. faecalis* from various matrices. This contrasts with the Enterolert-E test system, which could detect other enterococci, although the manufacturers do not provide data on the percentage of recovery for all enterococci species. The sensitivity of the RPA-LFA for the detection of *E. faecalis* in tap water, saline water, and in wastewater was 10–1000 times lower than that of Enterolert-E test, depending on the water quality.

The overall results confirm that the RPA-LFA developed was shown to be a specific and rapid technique that does not require major laboratory equipment and extensive training. This makes the assay potentially useful as a rapid assessment tool to confirm a sewer spill, when large numbers of *E. faecalis* are likely to be present. The assay can be used in remote settings with further research investigations and integration with molecular approaches [23,24,49]. A comparison between PCR, RPA-AGE, and RPA-LFA for the detection of E. faecalis is listed in Table 4.

### 3.4. Potential for RPA-LFA as a Quantitative Measurement System for the Enumeration of E. faecalis 

**Figure 6:**
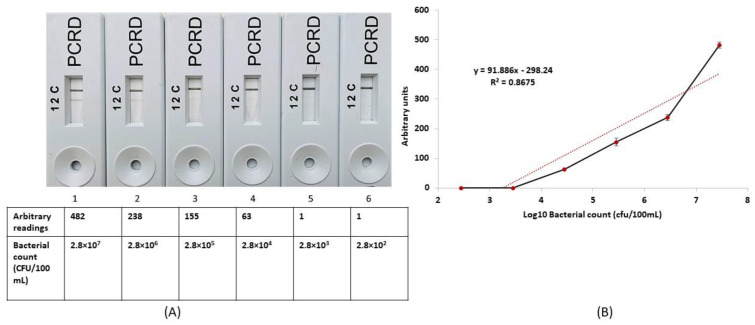
(**A**) Comparison of the RPA-LFA visual outputs and the values recorded by the GT810. (**B**) Standard curve plot of bacterial cell numbers v GT810 readings; (•) represents the value produced by the LF reads corresponding to the CFU count of bacteria per 100 mL of growth medium.

In a final experiment, the ability of the GT810 was determined to correlate the known number of *E. faecalis*) with the value readings given by the LF readed (Figure 6A). The results confirm a general correlation between actual cell numbers and GT810 output (Figure 6B), suggesting for the first time that RPA-LFA could be used for a quantitative assessment of enterococci present in water. However, the sensitivity of the RPA-LFA was not improved, as values of one were recorded on the GT810 at 2.8 × 10^2^ and 2.8 × 10^3^ organisms/100 mL. Therefore, the Creative Device LF reader does not enhance the sensitivity of the RPA assay, although quantitative reading could be obtained in the range 2.8 × 10^4^–2.8 × 10^7^ organisms/100 mL (Figure 6A,B). Because this RPA-LFA can quantify the presence of *E. faecalis* in wastewater and heavily contaminated environmental waters, it may have potential for use in a spill response setting, providing support to responders should there be large numbers of *E. faecalis* (2.8 × 10^4^/100 mL) present. However, future work would be required to increase the sensitivity of the assay and quantification.

## 4. Conclusions

Faecal enterococci are suggested as the superior FIB in water over coliforms because of their high density as a normal flora of the gastrointestinal tracts of animals and humans. *E. faecalis* has been found to be more prominent over other enterococci species. Therefore, detection of *E. faecalis* plays an important role to determine the faecal contamination in water and wastewater streams. Current methodologies to detect *E. faecalis* in environmental waters and wastewaters have numerous drawbacks, including the required time and need for sophisticated laboratory settings. Thus, this research aimed at the development of a rapid field test based on recombinase polymerase amplification coupled to a lateral flow assay (RPA-LFA) for the detection of *E. faecalis* in samples of surface waters and wastewater. The objective of this research was achieved with the 100% specificity of the assay for *E. faecalis*, detecting 2.8 × 10^3^–2.8 × 10^4^ organisms/100 mL in tap water, saline water, and wastewaters. The assay developed here could be completed in approximately 30 min using one constant temperature of 38 °C by persons with minimum training. In addition, this study also demonstrated the quantitation of *E. faecalis* using an LF reader for the first time, which enhances the potential use of RPA assay for enumeration of *E. faecalis* in wastewater and heavily contaminated environmental waters, surface water, and wastewater. However, the sensitivity of the RPA-LFA assay for the detection of *E. faecalis* in tap water, saline water, and in wastewater was 10–1000 times lower than that of the Enterolert-E test, depending on the water quality. Nevertheless, with further improvements, this low-cost RPA-LFA may be suitable to be used at the point-of-need (PON) if conjugated with a rapid field-deployable DNA extraction method. 

## Figures and Tables

**Table 1:** Primers targeting the genes *rpoB, rpoA,* and *tuf*, and probe used for recombinase polymerase amplification (RPA) coupled with lateral flow (LF).

Primer Name	Targeted Gene	Oligonucleotide Sequence (5′ to 3′)	Direction
Enttuf F1	*tuf*	TGACGATAAAGAAGAGAGCGGAGACACGAATC	Forward
Enttuf R1	*tuf*	CCCCATCTTTTTCATTTGGAGCGATAGTTTTT	Reverse
Enttuf F2	*tuf*	GACGATAAAGAAGAGAGCGGAGACACGAATCC	Forward
Enttuf R2	*tuf*	GCCCCATCTTTTTCATTTGGAGCGATAGTTTT	Reverse
Ent rpoA F1	*rpoA*	GCAGTGAAAACCGAAGCAAGCGCCATTCAAAT	Forward
Ent rpoA R1	*rpoA*	TAAGGCATCAAATTCTTCCTCCAATAAAATAT	Reverse
Ent rpoB F1 F1	*rpoB*	GGACCCGCTACCGTGACTGCCGGCGATATTATCG	Forward
Ent rpoB R1 R1	*rpoB*	Biotin-GAATCAACTGGAAGTACACCGATTGGCATATC	Reverse
Ent rpoB P1		**5′-FAM** -GATGTTGAGATCCTAAATAAAGATTTAGTTAT/dSpacer/TGTAGTGTTGCTGAAGGAG-**C3 spacer-3′**	Probe

**Table 2:** Estimated numbers of *E. faecalis* present in spiked tap water, saline, Koo Wee Rup wastewater, and Lang Lang wastewater, assessed using the Enterolert-E test (in organisms/100 mL).

Sample Dilution (from A Culture Containing 2.8 × 10^8^ Organisms/100 mL) Calculated Using Selective Isolation Plating. Actual Numbers are Shown in Brackets	Estimated Numbers in Spiked Tap Water	Estimated Numbers in Spiked Saline	Estimated Numbers in Spiked Koo Wee Rup Wastewater	Estimated Numbers in Non-Spiked Lang Lang Wastewater
Control (0/100 mL)	0	0	0	0
10^−6^ (2.8 × 10^0^)	0	0	1222.0–1986.3	0–1
10^−5^ (2.8 × 10^1^)	120–175	0	1630.4–2419.6	0.7–3.1
10^−4^ (2.8 × 10^2^)	1203–1750	20–30.4	>2419.6 to infinite	55.0–77.1
10^−3^ (2.8 × 10^3^)	>2419.6 to infinite	143.7–181.9	>2419.6 to infinite	126.1–187.2
10^−2^ (2.8 × 10^4^)	>2419.6 to infinite	>2419.6 to infinite	>2419.6 to infinite	120.8–179.3
10^−1^ (2.8 × 10^5^)	>2419.6 to infinite	>2419.6 to infinite	>2419.6 to infinite	126.1–187.2
10^0^ (2.8 × 10^6^)	>2419.6 to infinite	>2419.6 to infinite	>2419.6 to infinite	206.6–325.5

**Table 3:** A comparison of the advantages and disadvantages of current methods to identify and enumerate enterococci.

Technique	Description	Advantages	Disadvantages	Website
Selective Plate Isolation	Use of a dilution series of sample plated onto selective isolation media enabling the counting of colonies present (colony-forming units).	Simple detection system. Can be accurate.	Time consuming—24–48 h. Requires experienced laboratory technician and laboratory. Expensive in terms of consumables. May underestimate the number of enterococci in samples with high turbidity.	https://www.merckmillipore.com/AU/en/products/industrial-microbiology/culture-media/, (accessed on 20 November 2019)
Membrane Filtration	Use of membranes (pore size 0.22 −0.45 µm) to filter water after following by selective isolation plating (colony-forming units).	Efficient system. Can be accurate.	Time consuming—24–48 h. Requires experienced laboratory technician and laboratory. Expensive in terms of consumables. May underestimate the number of enterococci in samples with high turbidity.	www.oxoid.com/UK/blue/prod_detail/, (accessed on 20 November 2019)
IDEXX Enterolert-E	Based on the use of a defined substrate technology nutrient indicator to detect enterococci. This nutrient indicator fluoresces when metabolised by enterococci. (most probable number of cells).	No media preparation. Sensitive to 1 enterococcus per 100 mL. Less subjective interpretation. 50% fewer false positives and 95% fewer false negatives than the standard membrane filtration (MF) method.	Time consuming—24–48 h. Requires experienced laboratory technician and laboratory. Expensive in terms of consumables. Overestimation/underestimation of Enterococci is possible.	https://www.idexx.com.au/en-au/water/water-products-services/enterolert/, (accessed on 24 November 2019)
Polymerase Chain Reaction	Based on the use of 16S rDNA real-time PCR (gene copies).	Relatively fast (3–5 h), specific and sensitive. Multiplex PCR is possible. Quantitative detection (qPCR) of target pathogen is rapid (number of gene copies).	Well-equipped laboratory required. Requires primer optimisation and DNA extraction. Requirement of skilled personnel.	https://www.genesig.com/, (accessed on 4 December 2019)
Recombinase Polymerase Amplification	Versatile isothermal DNA/RNA amplification by TwistDx DNA polymerase. Based on the use of rpoB (RNA polymerase subunit B) specific gene target.	Rapid, less than 1 h.Carried out at 37–42 °C Requires very limited equipment and training.	Requires primer optimisation and DNA extraction.	http://www.twistdx.co.uk, (accessed on 21 September 2020)

**Table 4:** A comparison between PCR, RPA-AGE and RPA-LFA for detection of *E. faecalis*.

	Parameters	Sensitivity (CFU/100 mL)	Time Required	PON Applicability	Approximate Cost USD/Reaction	Temperature Required	References
Assay							
PCR		10^4^–10^5^	120–150 min	NP	5	Thermal cycling (95 °C, 55 °C, 72 °C)	[39,40]
RPA-EF		2.8 × 10^3^–2.8 × 10^3^	80 min	NP	5.5	38 °C	This study
RPA-LFA		2.8 × 10^3^	25–30 min	P	6	38 °C	This study

PCR: polymerase chain reaction; RPA-AGE: recombinase polymerase amplification-agarose gel electrophoresis; RPA-LFA: recombinase polymerase amplification-lateral flow assay; PON: point-of-need; NP: not possible; P: possible.

## Data Availability

All the data curated in this study are presented in the manuscript.

## Supplementary Materials

The following supporting information can be downloaded at: https://www.mdpi.com/article/10.3390/microorganisms11020381/s1, Figure S1: Specificity validation of Ent F1/R1 primer set. (A) RPA-AGE; Lane M: 100 bp molecular marker, Lane 1: non-template control, lane 2: empty (B) RPA-LF assay; LFA 1 – LFA 10: *E. faecalis*, *E. faecium*, *Staphylococcus aureus*, *Streptococcus pneumoniae*, *Bacillus cereus*, *Pseudomonas aeruginosa*, *E. coli* O157:H7, *Salmonella typhimurium*, *Shigella dysenteriae*, *E. coli* K-12; Figure S2: Sensitivity of RPA-LF assay for detection of *E. faecalis* using (A) Tap water; (B) Koo Wee Rup wastewater; (C) Lang Lang wastewater; (D) Saline. LFA 1 to 7: 2.8 × 10^6^ CFU/100 mL, 2.8 × 10^5^ CFU/100 mL, 2.8 × 10^4^ CFU/100 mL, 2.8 × 10^3^ CFU/100 mL, 2.8 × 10^2^ CFU/100 mL, 2.8 × 10^1^ CFU/100 mL, non-template control (NTC). Figure S3: Confirmation of the presence of *E. faecalis* in Lang Lang wastewater. LFA 1 to 9: RPA- LFA of Lang Lang wastewater neat, 10^−1^, 10^−2^, 10^−3^, 10^−4^, 10^−5^, 10^−6^, 10^−7^; and positive template control.
