# Supplementary material for: Development of A Rapid, Low-Cost Portable Detection Assay for Enterococci in Wastewater and Environmental Waters
Source: Microorganisms. 2023 Feb 2;11(2):381. doi: 10.3390/microorganisms11020381 (PMC9960780; doi:10.3390/microorganisms11020381)
Supplement: Supplementary file 1 [file microorganisms-11-00381-s001.zip › microorganisms-2126127-supplementary.pdf]

Supplementary Document

Development of a rapid, low-cost portable detection assay for enterococci in wastewater and environmental waters

Alka Rani Batra [1,2,3,*], Darren Cottam [3], Muriel Lepesteur [3], Carina Dexter [3], Kelly Zuccala [3], Caroline Martino [3], Leadin Khudur [1], Vivek Daniel [2], Andrew S. Ball [1] and Sarvesh Kumar Soni [1]

[1] ARC Training Centre for the Transformation of Australia's Biosolids Resource, School of Science, RMIT University, Bundoora West, Victoria 3083, Australia
[2] School of Science, RMIT University, Melbourne, Victoria 3083, Australia
[3] Environment Protection Authority Victoria, Centre for Applied Sciences, Ernest Jones Drive, Macleod Victoria 3085
* *Correspondence:* alka.rani@rmit.edu.au. Tel: +61399256594

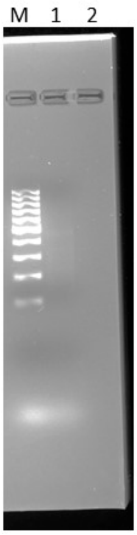

(A)

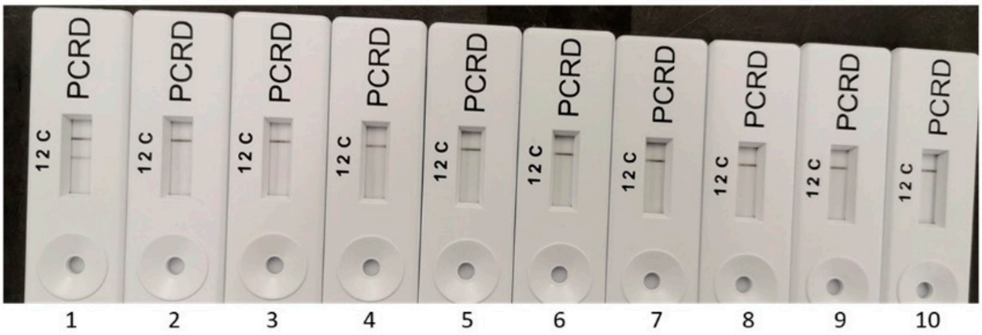

(B)

Figure S1: Specificity validation of Ent F1/R1 primer set. (A) RPA-AGE; Lane M: 100 bp molecular marker, Lane 1: non-template control, lane 2: empty (B) RPA-LF assay; LFA 1 – LFA 10: *E. faecalis, E. faecium*, *Staphylococcus aureus, Streptococcus pneumoniae*, *Bacillus cereus*, *Pseudomonas aeruginosa, E. coli* O157:H7, *Salmonella* Typhimurium, *Shigella dysenteriae, E. coli K*-12.

RMIT Classification: Trusted

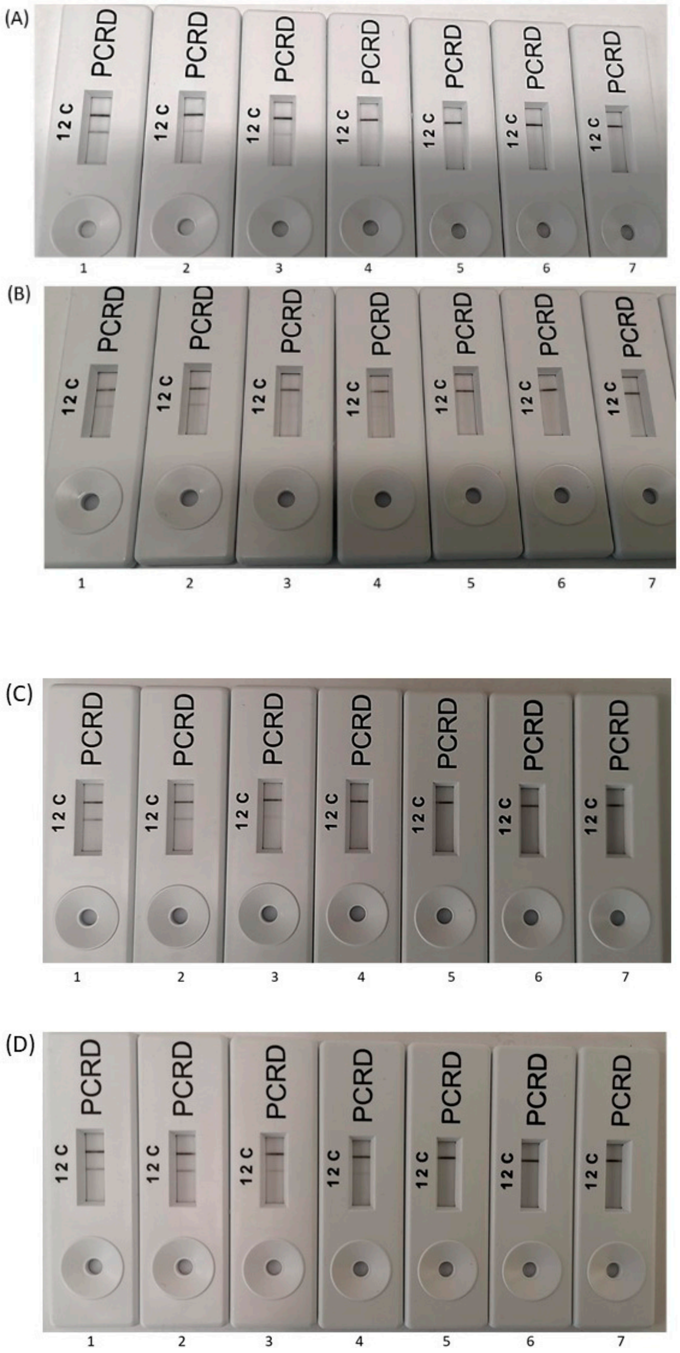

Figure S2: Sensitivity of RPA-LF assay for detection of *E. faecalis* using (A) Tap water; (B) Koo Wee Rup wastewater; (C) Lang Lang wastewater; (D) Saline. LFA 1 to 7: 2.8×10^6 CFU/100 mL, 2.8×10^5 CFU/100 mL, 2.8×10^4 CFU/100 mL, 2.8×10^3 CFU/100 mL, 2.8×10^2 CFU/100 mL, 2.8×10^1 CFU/100 mL, non-template control (NTC).

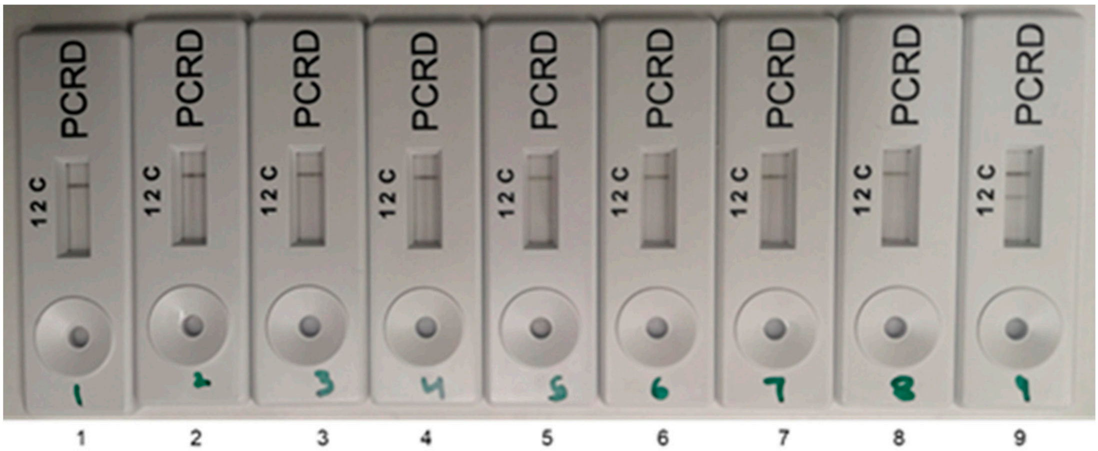

Figure S3: Confirmation of the presence of *E. faecalis* in Lang Lang wastewater. LFA 1 to 9: RPA- LFA of Lang Lang wastewater neat, 10^{-1}, 10^{-2} , 10^{-3} , 10^{-4} , 10^{-5} , 10^{-6} , 10^{-7} ; and positive template control.
